# The current status of stimuli-responsive nanotechnologies on orthopedic titanium implant surfaces

**DOI:** 10.1186/s12951-023-02017-8

**Published:** 2023-08-19

**Authors:** Jingyuan Han, Qianli Ma, Yanxin An, Fan Wu, Yuqing Zhao, Gaoyi Wu, Jing Wang

**Affiliations:** 1 Reconstruction and Regeneration, National Clinical Research Center for Oral Diseases, Shaanxi Engineering Research Center for Dental Materials and Advanced Manufacture, Department of Oral Implants, School of Stomatology, The Fourth Military Medical University, Xi’an, 710032 China; 2 School of Stomatology, Heilongjiang Key Lab of Oral Biomedicine Materials and Clinical Application, Experimental Center for Stomatology Engineering, Jiamusi University, Jiamusi, 154007 China; 3 Department of Biomaterials, Institute of Clinical Dentistry, University of Oslo, Geitmyrsveien, Oslo, 710455 Norway; 4 Department of General Surgery, The First Affiliated Hospital of Xi’an Medical University, Xi’an, China

**Keywords:** Nanotechnology, Nanostructure, Stimuli-response, Ti implant

## Abstract

With the continuous innovation and breakthrough of nanomedical technology, stimuli-responsive nanotechnology has been gradually applied to the surface modification of titanium implants to achieve brilliant antibacterial activity and promoted osteogenesis. Regarding to the different physiological and pathological microenvironment around implants before and after surgery, these surface nanomodifications are designed to respond to different stimuli and environmental changes in a timely, efficient, and specific way/manner. Here, we focus on the materials related to stimuli-responsive nanotechnology on titanium implant surface modification, including metals and their compounds, polymer materials and other materials. In addition, the mechanism of different response types is introduced according to different activation stimuli, including magnetic, electrical, photic, radio frequency and ultrasonic stimuli, pH and enzymatic stimuli (the internal stimuli). Meanwhile, the associated functions, potential applications and developing prospect were discussion.

## Introduction

Titanium (Ti) and its alloys are the most widely used metallic implantable materials for orthopedic defects treatment, on account of the excellent biocompatibility, low elasticity and great corrosion resistance [1]. However, in recent years, several drawbacks have been recognized in Ti-based implantable materials like insufficient osteogenic and antibacterial capabilities related with inherently bio-inert Ti surface [2]. Therefore, advanced surface characteristics related to physical, chemical and biological functions are crucial for improving the clinical performance of Ti implantable materials.

Surface properties modifications by biochemical coatings and morphological alterations on Ti-based materials have received enormous attention and became research hotpot over the last few decades [3]. The morphological modification technologies including chemical methods (e.g., acid/alkali etching, EA. etc.) and physical methods (e.g. ion implantation, physical vapor deposition, thermal spraying [4], etc.) provide different micro/nano surface structures on Ti and its alloys [5–7]. The absorption lacunae with diameters scales from 30 to 100 μm are formed by the acid secreted by osteoclasts to acid corrode the mineralized matrix and dissolve calcium phosphate crystals [8]. Microscale topographic modification mimicking these absorption lacunae dimensionally entails the cells containing these microscale grooves contact guidance physically for the sizes are similar to cells. Besides, nanoscale surface morphology is more attractive to actin cytoskeletal alignment and filopodia extensions of cells. Regarding to the in vivo environment where cells live inside extracellular matrix (ECM), cells are able to respond to environmental nanostructures via the interaction between their surface nanostructures (e.g. Receptors and filopodia, etc.) and nanoelements of ECM components (e.g. collagen fibrils, etc.) [9, 10]. The influence of such surface morphology on the process of bone integration has been reviewed in detail [11]. Nevertheless, these micro/nano-scale modifications mimicking bone structures and substrates applied on implant surfaces still have some limitations [12]. First, these modifications showed single function without the ability to respond to different physical environment changes like bone progenitor cells and immunocytes are constantly changing environments. In addition, drug release capabilities cannot be adequately synchronized or sequentially controlled, and their target-delivery of drug is also not ideal. For instance, non-directional drug release can lead to antimicrobe resistance of bacteria, and the change of the therapeutic dose cannot be controlled. These limitations would influence therapeutic effects, and even elicit other complications.

Stimuli-responsive nanomaterials have been widely investigated for their unique and intriguing properties have shown unique stimuli-responsive properties by adjusting the physical, chemical and biological properties of the materials to cause changes in properties or morphology for promoting implant bone integration ulteriorly. These materials can response the external stimuli effectively, including magnetic field [13–18], ultrasonic wave (USW) [19, 20], light [21–32], electric field [33–35], radiofrequency, pH [36–42], enzyme stimulus [43–47], or multiple stimuli [16, 27, 48]. After receiving the stimulating signals, the materials realize the on-demand controlled release of drugs, or produce photothermal (PTT), photodynamic (PDT), magnetothermal and other effects to achieve anti-infection, thereby promoting osteogenesis and other purposes.

In recent years, the stimuli-responsive nanotechnology of titanium implants has been reviewed. Hong et al. reviewed smart nanomaterials applied to biofilms formed/accumulated on orthopedic implants [10]. Ahmed et al. reviewed stimuli-responsive adaptive antibacterial implant biomaterials [49]. Montoya et al. reviewed antimicrobial applications of bioresponsive dental implant materials [50]. Zhang et al. reviewed triggered therapies from anodized nano-engineered titanium implants [51]. Similarly, Cai et al. reviewed stimuli-responsive TiO_2_ Nanotubes (TNT) drug delivery systems have been reviewed [52]. Jayaree et al. reviewed magnetic responsive materials in bone tissue engineering [53] etc. The existing reviews mostly concern single stimulus type or drug release mechanism with certain one-sidedness and limitations. At the beginning of this review, we described the commonly properties and applications of the commonly used stimuli-responsive nanomaterials, including elements and their compounds, polymer complex materials and other materials. Then, we firstly summarized different stimuli-responsive types and described the transformation principles of response related to titanium implant. Moreover, the biological effects applied on titanium surface were emphasized after the classifications, including antibacterial property and osteointegration. In the end, we spotlighted the potential research hotpot to provide a systematical reference for the development and translation in future research.

## Search strategy

Articles were identified to be included according to electronic search via, Embase, and Web of Science by using specific search strings revolving around stimuli-responsive titanium implant materials. The search yielded a total of 939 articles (PubMed 254; Embase 232; Web of Science 421; Additional records identified through other resources 32). After removing duplicates, 740 articles were retrieved. After reading the titles and abstracts, 63 records were retrieved. The full-text articles were further assessed for eligibility and a total of 59 studies could be included in this review.

## Types of nanomaterials

Stimuli-responsive nanomaterials on the Ti implant surface include metal materials, high polymer materials and other materials. Metal materials mainly include Ag, Fe, Au, Zn, Cu, Mo, Ti and their compounds. Polymer complex materials include modifiable liposomes, chitosan (CHI), micelles, hydroxyapatite, etc. mainly react as shell of nanoparticles with drugs or other materials inside.

### Metal and metal alloy

Metal nanomaterials on titanium implant surface attract lots of attention in the biomedical field for their inert nature and intrinsic characteristics. Noble metal nanoparticles (such as gold and silver, etc.) have unique surface isoionization resonance phenomenon, which can enhance their radioactive absorption and scattering properties. Researchers changed these features by altering attributes such as size/shape of their basic unit, and functionalization properties [54]. Despite ideal stability, metal materials exhibit relative high cytotoxicity and are easy to accumulate in organs, eliciting adverse effects in host tissue [55]. Therefore, biocompatibility and metabolism should be considered in the experimental design.

#### Ti

TiO_2_ Nanotubes (TNT) are widely used on Ti implant surface study, which could be simply fabricated by electrochemical anodization (EA) on regular or irregular pure titanium surface [51, 56]. The dimension of TNT can be controlled by EA voltage/time. Adjustable one-end closed tubular structures are beneficial for loading medicine inside to establish drug sustained-release system [57]. Besides, TiO_2_ also has unique light and ultrasonic responsive properties, which can be used to design antibacterial and promote bone experimental studies [58].

Regarding to that TNT is an ideal platform and drug carrier for drug storage and release, several studies have applied various stimuli-responsive coatings (biological, chemical, electrical, magnetic) to TNT-based drug delivery systems (DDS) which is the widely used in titanium implant research [59]. Magnetite hybrid nanocomposite-loaded TNT is used to treat infection [13]. Au nanoparticles (AuNPs)-loaded-TNT response radiofrequency (RF) is used to control drug release [60]. Indomethacin (Ind) micelle loaded-TNT response USW controls local drug release [19], etc.

The crystal structures of TiO_2_ have important effects on the photocatalytic performance, mainly divide into anatase and rutile crystal form. It is generally believed that the former has higher photocatalytic activity which has been used as photosensitive coating [61]. The difference is reflected in the degree of distortion of the octahedron and the way of interconnection between the octahedrons. In addition, ultrasonic can assist the photocatalytic ability [48]. Ultrasonic vibration and microjet can improve the effect of mass transfer between solid photocatalyst and liquid interface. Therefore, TiO_2_ can produce electron–hole effect under the action of light or ultrasonic assisted photocatalysis under light irradiation and USW stimulation, which can generate highly reactive free radicals and produce reactive oxygen species (ROS) to kill bacteria. The ROS can destroy a variety of important biological polymers and membranes in the cell, but also can form other active oxidation substances, which is very averse to the continued growth and reproduction of bacteria, so as to play an antibacterial role. But the photocatalytic activity is limited by the band gap of TiO_2_ entails that TiO_2_ can only accept the light whose wavelength ranges in the ultraviolet spectrum [26]. Several elements have been doped in TiO_2_ to solve this problem. For example, Zhang et al. [62] reported that, incorporating F, Yb, and Ho to TiO_2_ nanorods could improve the photocatalytic ability and eradicate single species biofilms like *Staphylococcus aureus (S.aureus)* and *Escherichia coli (E. coli)* through a combined mechanism of PTT effects, PDT effects, and physical destruction. Moon et al. [25] deposited the Au and Pt nanoparticles on anodized 100 nm TNT by ion plasma sputtering which extend the limited photocatalytic effect of TiO_2_ in the ultraviolet to visible light region.

#### Fe

Ferric oxide is the most widely used magnetic-responsive material. It’s a simple and effective way to give materials high magnetic properties by introducing ferric oxide into biological substrates, and it’s easy to control the magnetic strength related to ferric oxide content [53]. Researchers combine magnetic ferric oxide with different nanoparticles or implant surface coatings to achieve the purpose of targeted antibacterial and osteogenic stimulation by using its unique magnetic response properties. Surface modifications, such as polyethylene glycol (PEG) [15, 63], dopamine (DOP) [64], hydroxyapatite [65] and hydrogels [63], can improve the biocompatibility, bioactivity, hydrophilicity/hydrophobicity and drug loading stability of ferric oxide nanoparticles. In addition, ferric oxide nanoparticles have PTT properties and magneto-thermal effect are important aspects of its anti-infection applications [66, 67].

#### Ag

With the great progress of nanomaterials and nanotechnology, the effect of Ag nanoparticles (AgNPs) against broad-spectrum infections has been significantly improved in the stimuli-responsive nanomaterials [68]. AgNPs have high reactivity due to their quantum size effect and high specific surface area and have very strong antibacterial effect by irradiating AgNPs at resonant wavelengths to lead to local temperature rise and PTT. By surface functionalization (e.g., biomolecular and ligand binding), nanoparticles can selectively target specific abnormal cells and produce an effective thermal gradient on the cell, thereby affecting cell activity and integrity [31]. The surface plasmon resonance of AgNPs can also be influenced by size, shape, surface coating, and solution chemistry. These changes can affect the PDT antibacterial efficacy. [31]

However, AgNPs might accumulate in the reticuloendothelial system and induce the genotoxic and cytotoxic damage of human lung fibroblasts and glioblastoma cells by disrupting the cell membranes lead to hepatic and renal injuries [56, 68–70]. High concentrations of AgNPs may inhibit osteoblast proliferation and decrease implant survival [71]. Therefore, AgNPs often combine with other nanomaterials to produce synergistic and complementary effects in the stimuli-responsive system [31]. It is necessary to ensure its safety use in further bone-related applications.

#### Zn

Zinc is an important trace element of many enzymes and proteins with good chemical stability and low toxicity to human cells. Zinc maintains cell membrane structure and plays an important role in normal biological activities tracellularly, such as DNA synthesis, enzyme activity control, and cell apoptosis control [72–74]. Zinc oxide nanoparticles (ZnO-NPs) are commonly used due to their low toxicity, good biocompatibility, and chemical stability [73]. In neutral to acidic environment, ZnO NPs exert their antibacterial functions mainly via the release of ROS and Zn^2+^ [75]. The pH-sensitivity of ZnO quantum dots (QD) and ROS production have also been applied in the research field of stimuli-responsive nanoengineering [39]. It is worth noting that excess ZnO-NPs can alter cell morphology, damage DNA induce genotoxic effects and destroy cellular defense systems, leading to cell apoptosis or necrosis finally [75, 76]. Hence, ZnO-NPs are often combined with other biomaterials to obtain a variety of biofunctions and corrosion resistance.

#### Cu

Cu is the basic mineral of many proteins and enzymes with excellent biological activity and antibacterial ability and [77]. Similar to AgNPs, the antibacterial ability of Cu nanoparticles (CuNPs) is interrelated to the production of ROS and Cu^2+^. In stimuli-responsive nano systems, after being modified by ammonia, CuNPs could be established as pH-responsive nanomaterials on TNT to promote vascularization and osteogenesis [36]. In addition, pH responsive CuNPs can also be used on PEEK implant [38]. In addition, Cu promote collagen maturation and further induce osteogenic differentiation of mesenchymal stem cells through lysine oxidase crosslinking [78]. CuNPs have the optical properties of plasmon resonance and the optical response ability of absorbing fluorescence. Its ability of producing optical effects can be considered in future studies [82].

#### Mo

Molybdenum alloy coatings have been widely used for their photoresponsive performance. As a transition metal dihalide similar to graphene, MoS_2_ has significant antibacterial properties and shows high PTT conversion efficiency for two-dimensional ultra-thin atomic layer structure and high specific surface area [79]. MoS_2_ is always formed as nano-coatings combined with polydopamine (PDA)–arginine–glycine–aspartic acid (RGD) [80] and CHI-modified MoS_2_ coating on the surface of titanium implants [81]. These coatings all showed good bactericidal and osteointegration promotion effects under 808 nm NIR. Besides, MoSe_2_ has also been incorporate into studies in recent years which can improve the light-responsive capability of TiO_2_ prepared by micro-arc oxidation to be activated under NIR [21]. As a metal substance with high cytotoxicity, Mo has been combined with CHI, protein and other substances to improve biocompatibility.

#### Au

With the advantages of simple synthesis, robustness, inertia, good biocompatibility, controllable geometric and optical properties [82], AuNPs could be modified to carry drugs, peptides, proteins, DNA, RNA and other materials to achieve better functionality [83]. In stimuli-responsive studies, RF energy [60] and NIR [32] could be efficiently absorbed by AuNPs. The generated heat is released to the tissue surrounding to boost new bone formation efficiently and [84, 85]. The optical response of AuNPs has also been applied to remove the biofilm on implant surface [86]. However, AuNPs can be phagocytosed by cells, leading to apoptosis or necrosis, and even accumulate in organs [83].

The metal particles of the implant surfaces will be released due to controlled releasing, wear and tear, chemical degradation, etc. [87]. High concentrations of local or systemic metal particles can lead to some adverse effects, such as metal autoimmunity, disintegration or bioaccumulation in the body, and systemic toxicity [88]. After the metal particles are endocytosed into the acidic and enzyme-rich cytoplasm, they will be degraded and release overdosed metal ions [89]. High concentration metal ions can modify subcellular organelles and physiological functions through various mechanisms like affect gene/protein expression, damage cell membrane, disrupt electron transport in mitochondrial intima, and then produce endogenous reactive oxygen species, and so on [88, 90–92]. Additionally, metals and their oxides can stimulate major signaling pathways nuclear factor (NF)-κB, and mitogen-activated protein kinase (MAPK), and then generate proinflammatory effects through interactions with the immune system cells [93]. The toxic effect of metal particles may be closely related to the shape, size, dose, and some other physical properties of the particles [94, 95]. For example, larger metal particles are not easily metabolized, while smaller particles are easily degraded but also enhance their cytotoxicity. The stimuli-responsive properties of metal materials can effectively realize the drug-controlled release effect, photothermal effect, photodynamic effect, magnetodynamic effect, magnetothermal effect, acoustic dynamic effect etc. [31, 32, 60, 65]. In orthopedic implants can achieve good antibacterial (overcome bacterial resistance to antibiotics), promote vascular neural network regeneration, promote bone regeneration and other capabilities [71, 96, 97]. Based on the properties, exposure pathways, uptake and metabolism, and toxic effects of the metal materials, the strategies to reduce or eliminate the adverse health effects of metals/NPs should be taken.

### Polymer materials

#### Liposome

Liposome is a kind of molecular ordered multilayer vesicle structure assembly formed spontaneously in water by association of phospholipid and water. Each layer is a lipid bilayer composed of phospholipid and cholesterol which is similar to the biological characteristics of cell membrane [98]. It can used as drugs (water-soluble and fat-soluble drugs) delivery which can prolong the half-life of drugs [99]. In stimuli-response nanotechnology field, it can be modified by different molecules on the surface to respond to pH, enzyme, NIR, USW and some other stimuli. After receiving these stimuli, the liposomes disintegrate and release the encapsulated drugs, so that/achieve well-controlled drug release. However, there are still some limitations during the use of liposomes, short time circulation in vivo, weak active targeting and poor stability [100].

#### Micelle

Micelles consist of a core of hydrophobic groups and an outer layer of hydrophilic groups. The hydrophobic parts of many surfactant molecules attract each other and associate together to form micelles and form various shapes, such as spherical, layered, rod. Micelles have high drug loading efficiency, wide drug loading range, good stability, long retention in vivo, and can be modified to respond to environmental changes sufficiently [101]. In the stimuli-response nanotechnology of Ti implant surface, micelles can be applied to respond pH [37] and enzyme [102] response ability to achieve ideal drug targeting and specific release property. Besides, micelles are also designed to respond to other types of stimuli, such as optical [103], temperature [104], enzyme, USW, oxidation, etc. [105].

#### Chitosan

Chitosan is an aminopolysaccharide from the exoskeleton of crustaceans and can be commercialized by deacetylation. The degree of deacetylation (D.D) determines the content of amine group (NH_2_) in the macromolecular chain. Moreover, the increase of D.D leads to the charged groups increase of CHI in dilute acid solution and the charge density increase of polyelectrolyte due to the decentralization of amine matrix. Its chemical structure is a cationic polymer alkaline polysaccharide polymer with unique physical and chemical properties and biological activation function. It is a carrier material that is biodegradable, safe, biocompatible, easy to modify and obtain, cheap, hydrophilic, pH reactivity etc. [106]. Surface modification can make it have good stimuli-response performance, such as magnetic [107] and electrical [34]. These properties make CHI an ideal carrier for adding antibiotics on titanium surface and have great research prospects for stimulus-responsive nanotechnology. CHI can achieve well controlled drug release by modifying its surface. CHI has a stable structure, and it is difficult to accept stimulative signal to release of internal drugs without modification.

#### Hydrogel

Hydrogel is a polymer network system formed by stable chemical or physical crosslinking of hydrophilic polymers. Physical crosslinked hydrogels are hydrogels formed by electrostatic force, hydrogen bond, hydrophobic interaction and other intermolecular force crosslinking. This kind of hydrogel has low mechanical strength and will change into sol when the temperature rises. Chemical crosslinked hydrogels refer to gels that crosslink polymers into networks via covalent bonds. Among them, the covalent bond is generated by the “click” reaction, such as mercaptan–ene/friend central addition, mercaptan–epoxy reaction, azide-acetylene cycloaddition, Schiff base reaction, epoxy-amine reaction, mercaptan–disulfide exchange reaction. Many advantages like swelling, flexibility, property of easy to be modified, good biocompatibility entail the hydrogel widely used in carrier materials in drug delivery system. Hydrogel can load a variety of bioactive compounds, such as hydrophilic and hydrophobic drugs, proteins, peptides, fluorescent molecules, etc. [108]. Hydrogels stabilize in vivo without pretreatment. Drug release rates can be appropriately controlled by changing the composition of hydrogels [109]. After modification they can response different stimuli like temperature [110], light, electric, pH, enzyme, USW, etc. in tumor and orthopedic diseases fields [111]. Under the action of external stimulation, the internal molecules of the hydrogel open crosslinking and the hydrogel gradually degrades to achieve the drug slow-release effect, which can be designed to match implant bone integration with long healing time.

### Other materials

#### P

Phosphorus is an important element in bone tissue and accounts for 1% of body weight, high PTT capacity. The degradation products are phosphate and phosphate esters with good biocompatibility [112]. Red phosphorus is used to modify the surface coating of titanium implants, which produces PTT effect under NIR and can eradicate the biofilm on the implant surface. Tan et al. [29] prepared a P coating on Ti which responses the NIR to produce PTT effect, and then achieve good antibacterial and osteogenic effect finally. However, as light-responsive material, the problems of burning normal tissues and structural instability should be considered carefully in the clinical application [113].

#### GO

Graphene oxide (GO) has attracted much attention due to its large percentage of surface area, good water solubility and biocompatibility. Due to the conjugated sp [2] structure, GO is prone to fluorescence resonance energy transfer effect with fluorescent molecules, resulting in fluorescence quenching [114, 115]. In addition, GO can interact with osteoblasts and stem cells to promote vascularized bone fusion. Some studies utilized GO as the carrier of AgNPs for GO is favorable for the adsorption and distribution of AgNPs [116]. Xie et al. reported a GO/Ag/collagen coating on Ti which responses 660 nm visible light to produce ROS to achieve antibacterial effect [31]. However, the preparation procedure of GO is complex, requiring expensive equipment and harsh preparation conditions [117].

### Composition of nanomaterials

Different types of nanomaterials can be composed to exploit their advantages and cover defects to achieve multifunctional effects. Stimuli-responsive nanostructures on Ti implant can be classified as composite nanoparticles and composite nano-coatings.

#### Composite nanoparticles

**Fig. 1:**
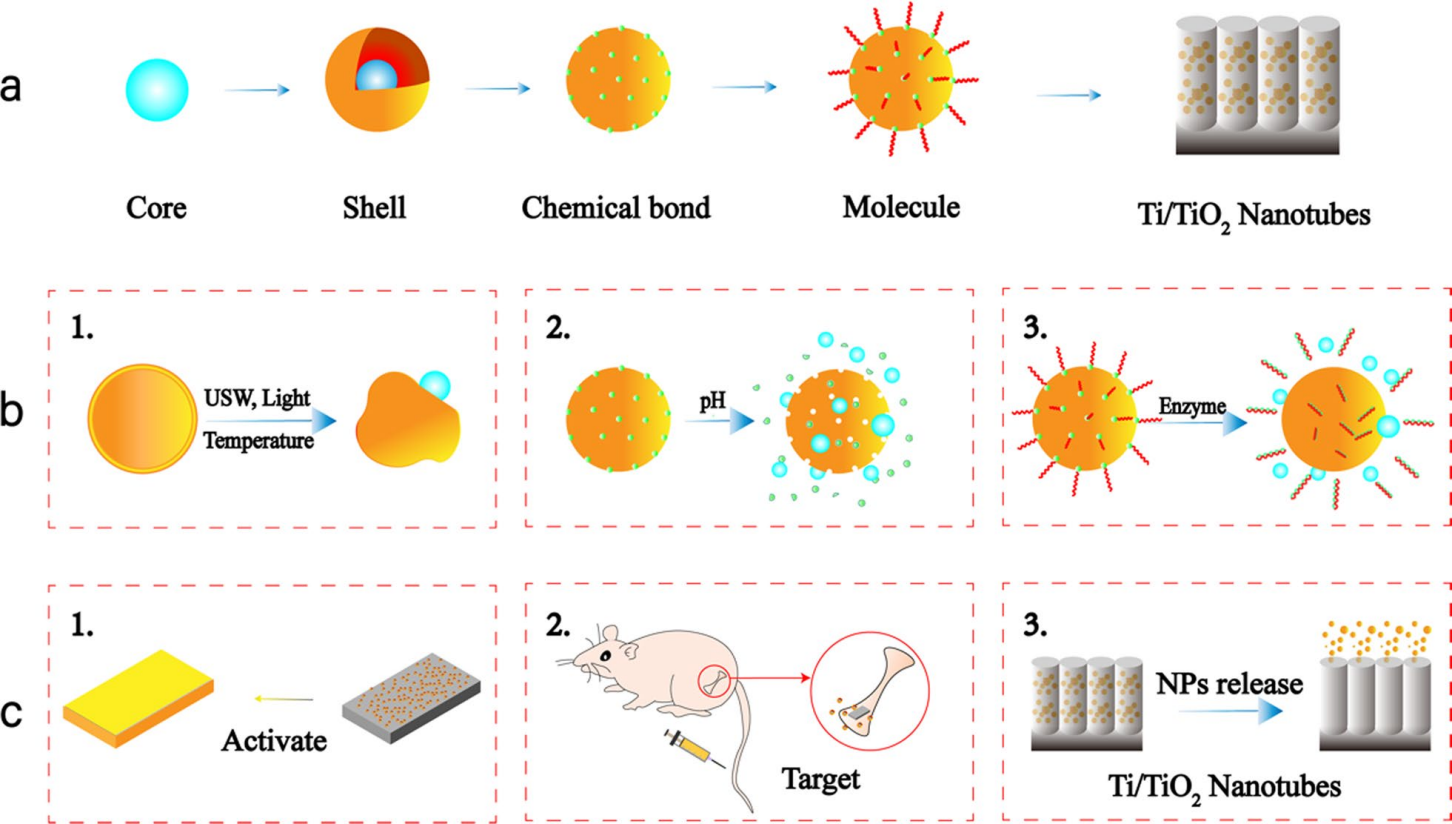
Nanoparticle preparation and drug release. **a** The process of nanoparticle preparation: (1) the core of the NPs could be drugs or other metal materials like Fe, Au, Ag, etc. (2) the shell of the NPs could be hydrogel, chitosan, micelle, liposome, CHI, etc. (3) the chemical bond on the shell could be disulfide, hydrazone bond or other bonds which connect the shell and molecules outsides. (4) the molecule which modify the NPs can improve the performance typically. **b** Stimuli could be designed on any part of NPs: (1) after accepting the USW or light, the shell break to release the drug inside. (2) After receiving the signal of pH change, the bond break or convert to release the drugs inside. (3) The molecules combining with enzyme from bacteria or osteoclast release drugs inside. **c** The NPs could accept the stimuli like MF, RF and light which achieve the purpose of targeting, active, or NPs releasing aim

Most stimuli-responsive nanoparticles are core–shell structures with surface modifications of different molecules to improve biological properties. The commonly used shell materials for titanium implants include CHI, hydrogel, micelle, liposome, and mesoporous silicon. Different drugs or stimuli-responsive materials are loaded as cores inside. Stimuli-responsive points can be designed in any part of core–shell structures, including the shells, the cores, the bonds, and even the molecules (Fig. 1). The core which could always accept the stimuli like magnetic field and RF which achieve the purpose of targeting and active aim [60, 118]. After accepting the USW, the shell break to release the drug inside [98]. PH-responsive chemical bond break or convert to release the drugs inside in infected acidic tissue [41]. The enzyme-responsive molecules could combine with enzyme from bacteria or osteoclast which can be used for targeted therapy and controlled drug release [47]. Hydrogels can be expanded and released at high temperatures, or they can be designed to concentrate at high temperatures and split at room temperature [110]. Micelles can release internally loaded drugs in response to pH [37]. Mesoporous silicon surfaces can be modified with disulfide bonds in response to acidic pH to release of internal loaded drugs. Nanoparticles with iron oxide as the core can respond to the external magnetic field to achieve targeted drug delivery [119]. Chi-Ag-MoS_2_ nanoparticles can perform targeted and rapid removal of biofilms under light stimulation and have good biocompatibility [81]. Stimuli-responsive drug delivery system is widely used in the field of biomedicine, and its application in titanium implants is still promising.

### Composite coatings

**Fig. 2:**
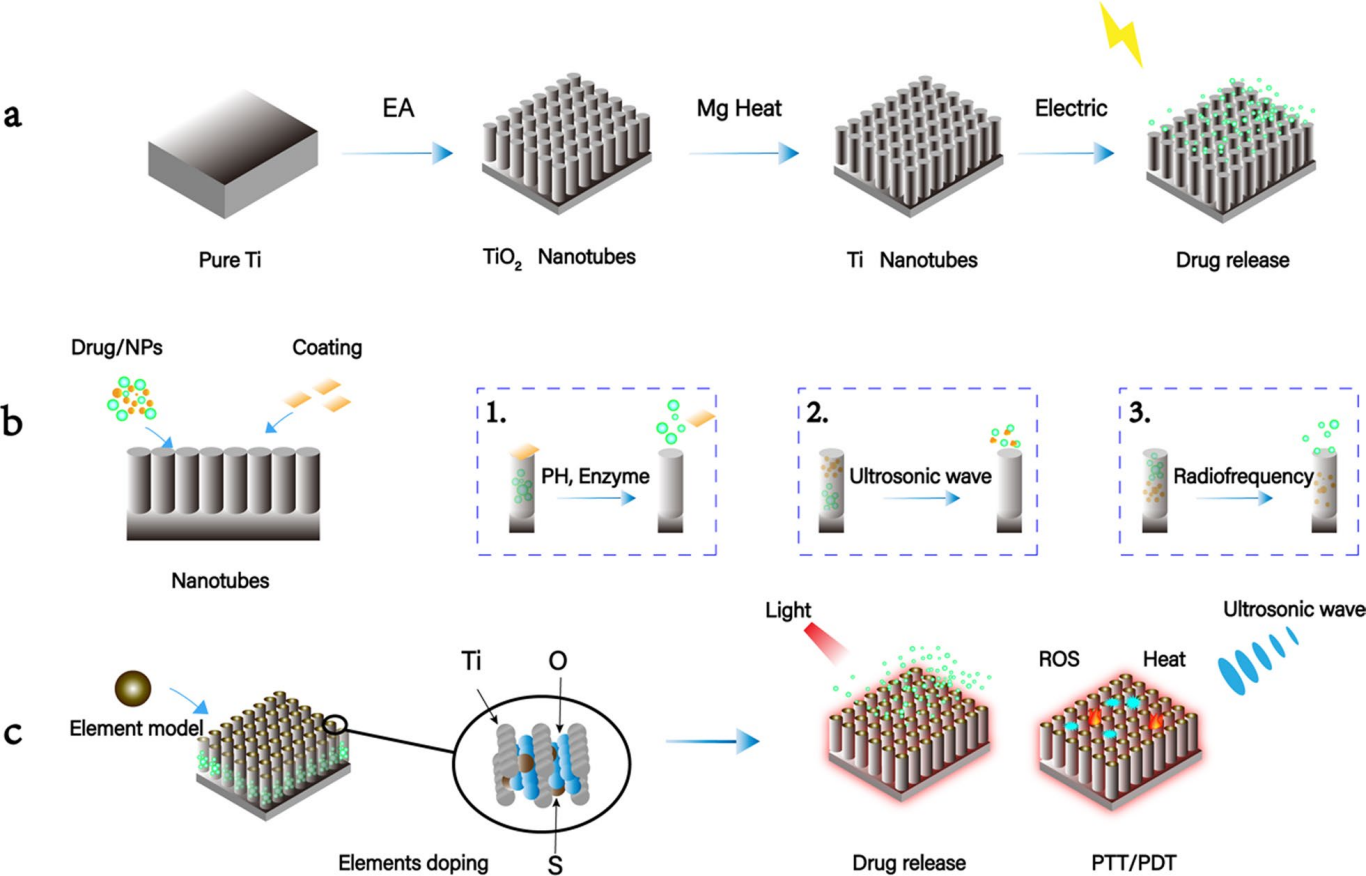
Stimuli-responsive nanotechnologies on TNT. **a** Preparation of TNT by EA, which could only accept the light which wavelength ranges in the ultraviolet spectrum [26]. According to mg-heat treatment, TiO_2_ nanotubes were converted into Ti nanotubes to response electric stimulus and release the drug inside [35]; **b** loading coatings on nanotubes and drugs or nanoparticles inside is the most common application of TNT. There are different coatings to seal the nozzles, like which could response pH [39] and enzyme [43], after receiving the stimuli, the coatings open and contents flow out (1); and loading nanoparticles above the drugs, and release or break after receive the stimuli, for example liposome-rhBMP-2 nanocomplexes which respond USW, and then the liposome break to release the rhBMP-2 (2) [19]; the strategy that nanoparticles loaded on the bottom of TNT with drugs loaded above is another drug delivery way, in which the active nanoparticles could push the drugs out of the TNT (3) [60]; **c** elements doping such as S, N, metal, etc. could improve the photoresponsive ability of TNT

The most widely used method for surface modification of Ti implants at present, refers to coating with non-single component on Ti by layer-by-layer (LBL) self-assembly [43], micro-arc oxidation (MAO) [57], element doping [26], preparation of hydrophobic surfaces [20], successive ion layer adsorption and reaction (SILAR) [120], magnesiothermic reduction process [35] and other methods. Tan et al. replaced O atoms in TiO_2_ with S to prepare S-doped Ti–S–TiO_2-x_ coating with ultrasonic response and catalytic PTT properties [48]. Yu et al. prepared TNT-DFO-HA-Gen coating with HAase enzyme response ability by LBL, showing extremely high enzyme sensitivity and specificity to kill bacteria and remove biofilms [44]. (Fig. 2).

## Types of stimuli-responsive strategies

### Magnetic responsive strategy

Magnetic-responsive nanotechnology refers to the magnetic field influences directly or indirectly on the tissues around the magnetic materials and affect the biological behavior. In addition, magnetic-responsive materials can response to the external magnetic field to achieve targeting effects and magnetocaloric effects. Magnetic response nanotechnology has been widely used in the research of anti-infection and osteogenesis of implants due to its high magnetic response performance and biological properties of magnetic field [53, 119, 121].

Magnetic field can promote bone-implant integration, accelerate healing of bone fractures and increase calcium content [122] by providing the mechanical stimuli which could be sensed and responded by the cells [123]. Bariana et al. [66] showed that the significant cell behaviors changes like alteration of cell membrane and extracellular matrix proteins under the externally applied magnetic fields including the static magnetic fields (SMF) and pulsed magnetic fields (PMF) [63].

SMF accelerate osteogenic differentiation of rat osteoblasts [124], human osteoblast-like MG63 cells [125], adipose-derived mesenchymal stromal cells (ASCs), adipose-derived stem cells (ADSCs) [126], human bone marrow-derived mesenchymal stem cells (BMSCs) [127], mouse embryo osteoblast precursor cells (MC3T3-E1) [128], pre-osteoblast cells [129], and stromal vascular fraction cells (SVFs). These cells are potent primary cell sources to produce highly vascularized bone graft substitutes [63]. In addition, the adhesion and differentiation of osteoblastic cells and the bone formation could be increased by adding magnetic nanoparticles (MNPs) to biopolymer scaffolds [130]. PMF also show a positive effect on the differentiation of cartilage cell [131], Chondrocytes, osteoblasts [132], BMSCs and ADSCs [132, 133] etc. Compared with the low frequency PMF, most studies proved that the high frequency PMF inhibit cell proliferation and destroy the related genes [134]. Different from SMF, PMF regulate cell cycle and secretion of cytokines affect the signal transmission on the cell membrane [135]. The magnetic field can also improve the synthesis of bone morphogenetic protein-2 (BMP-2) which is one of the main endogenous growth factors to induce osteogenic differentiation and promote calcification [136]. Additionally, studies show that the magnetism can be utilized to cells according to magnetic scaffolding materials without applying the external magnetic [137, 138]. Yang et al. presents that the paramagnetic implants can enhance the osteogenic response of pre-osteoblast cells [17]. The magnetic nanoparticles acting as magnetic actuation can facilitate osteoblast differentiation and promote mineral deposits of stem cells.

The external magnetic field can penetrate human tissue and control the magnetic nanomaterials. This unique response capability is the basis for the application of magnetic-responsive nanomaterials in the biomedical field, which allows them to reach the target site for targeted delivery and control drug release under the magnetic field [14]. The targeting modes of magnetic-responsive nanomaterials mainly shows in two ways. Firstly, in the presence of an external magnetic field, the drug-loaded nanoparticles are delivery to the target position. Secondly, magnetic drug-carrying nanoparticles were preloaded on the implant and their release was controlled by magnetic field.

In recent years, magnetic targeting on Ti has been studied as follows. Li et al. [139] aiming at the weak integration of titanium and skin, prepared Fe_3_O_4_ nanoparticles superparamagnetic TiO_2_ coating by micro-arc oxidation method which can effectively prevent soft tissue decay and inflammatory reaction. Janßen et al. [14, 15] synthesized super-paramagnetic CHI core–shell nanoparticles and modified them with fluorine carriers (fluor thiocyanate/FITC or rhodamine B isothiocyanate/RITC) and PEG. Combined with magnetic metal plates in vivo and magnetic fields in vitro, showed super-paramagnetic high porosity and good biocompatibility, which is an ideal magnetic drug targeting model. Yang et al. [17] aimed at the problems of AgNPs loss and cytotoxicity on the surface of dental implants, designed magnetic nanosystem. Ag–Fe_3_O_4_ nanoparticles were coated in TNT and permanent magnet was loaded in implant to adsorb nanoparticles, which showed good antibacterial activity.

Some studies also applied the magnetic nanoparticles response principle to TNT drug delivery system, but only used in vitro. Aw et al. encapsulated Fe_3_O_4_ nanoparticles modified by dopamine at the bottom of TNT, with polymer micelles as the carrier above to wrap Ind [13]. In this study, three amphiphilic polymer micelles with different properties and sizes, d-α-tocopherol succinate 1000 (TPGS), Pluronic F127 and PEO-PPO-PEO, were selected to demonstrate good magnetic drive drug release system. Shrestha et al. embedded magnetic materials into TNT which can not only decompose organic matter by magnetically guided thermal catalyst, but also release active drug model by using TiO_2_ photocatalytic effect (Table 1).

**Table 1:** Magnetic responsive nanotechnology designs

Response system	Magnetic field type	Preparation processes	Responding mechanism	Drug delivery	Main results
Magnetically responsive TNT delivery system (Aw2012 [13])	External permanent magnet	1. TNT was prepared through EA 2. Dopa-Fe_3_O_4_ magnetic nanoparticles are loaded at the bottom of TNT 3. Micellar coated Ind was loaded on the upper end of magnetic nanoparticles	Magnetically drives magnetic nanoparticles from the bottom	Preloading drugs in TNT	Achieve drugs-controlled release of magnetic response
MNPSNPs (Janßen2018 [14])	EM field/1.8 T/10 min	1. Fe_3_O_4_ is loaded with mesoporous silica 2. Fluorescein FIEC and RIBC are attached to the surface 3. Peg-silane modification 4. Nanoparticles are loaded onto Ti surface	Electromagnetic response property of Fe_3_O_4_	Tail vein injection	1. Good drug targeting 2. Good biocompatibility
Superparamagnetic PLGA coating (Ag–Fe_3_O_4_) (Yang2018 [17])	Permanent magnet is embedded in dental implants	Ag–Fe_3_O_4_ nanoparticles are loaded with PLGA coating on Ti surface	Magnetic adsorption magnetic particles; antibacterial activity of Ag ions	Nano-coating	1. The fixed PLGA 2. Good antibacterial activity 3. Promote the proliferation of bone cells
Superparamagnetic TiO_2_ coating (Li2019 [139])	–	Fe_3_O_4_ modified TiO_2_ coating	Effect of micromagnetic field on fibroblast	Nano-coating	1. Inhibit bacterial adhesion and reproduction 2. Improve soft tissue integration

Although magnetic-responsive materials have been widely studied in Ti implant, there are still some unavoidable problems. Firstly, magnetic nanoparticles have small size, poor stability, and low magnetism, so that agglomeration always occur in the synthesis and use process, which affect the function. Secondly, due to the complex physiological environment in vivo, the cumulative safe dose threshold and side effects in vivo are still unclear [140]. Moreover, specific duration and strength of magnetic field stimulation can inhibit.cytokine secretion, proliferation and differentiation [53]. Compared with pH, enzyme response and other stimulation responses, external magnetic field instruments may be needed. Furthermore, many experiments and clinical practices have shown that the influence of magnetic field on the body is related to the intensity of magnetic field, type of magnetic field, direction of magnetic field and time of action [53]. But there are lots of controversies about the most suitable physicochemical properties of the magnetic response materials for human body, such as surface charge.

### Photoresponsive strategy

Photoresponsive nanotechnology is the process of applying specific wavelengths of light (such as visible light, ultraviolet light, infrared light) to illuminate nanomaterials TNT [32], P [29], MoS_2_ [81], etc. respond to the light to produce effects, so as to achieve antibacterial therapeutic or promote osteogenesis. Photoresponsive nanotechnology has the advantages of small tissue invasion, deep tissue penetration, immediate treatment effect, and improvement of bacterial drug resistance [23–41]. The effects of photoresponsive nanotechnology mainly involves PTT and PDT effects [29].

**Fig. 3:**
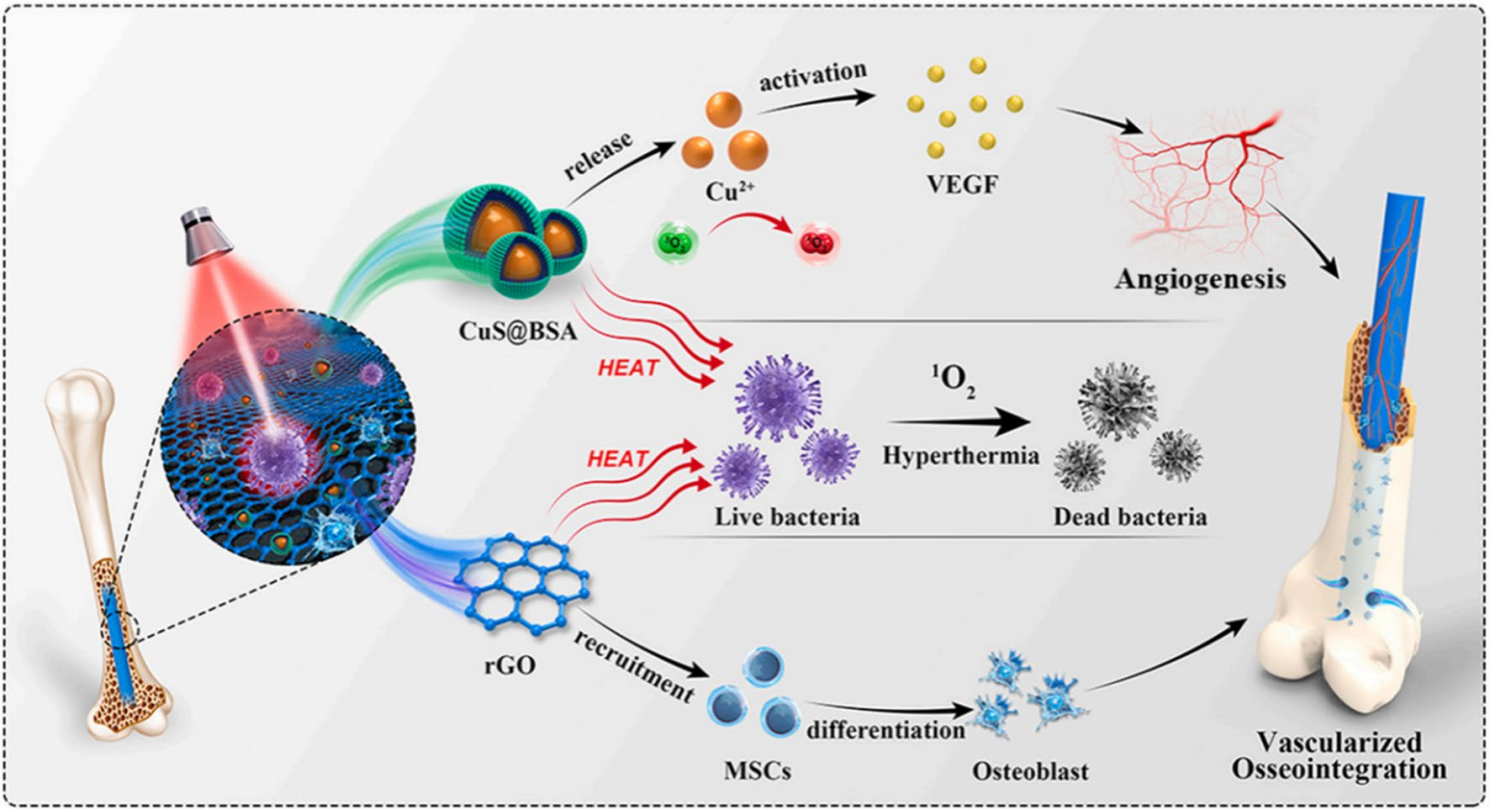
Schematic illustration of synergistic photocatalytic antibacterial and osseointegration via coupling CuS@BSA NPs and rGO without biologics. Copyright 2017, Biomaterials

PTT effect refers to PTT agent kills bacteria and eradicates biofilms by thermally induced therapy under light, which is the most common effect in photoresponse research (Fig. 3) [141]. PDT effect refers to O^2^ is produced in the process of PDT, which improves the thermal sensitivity of biofilm by increasing the permeability of bacterial cell membrane and cell wall [142]. In addition, PTT and PDT effects can be synergistic. Photoresponsive materials exist at nanometer scale, and have enhanced surface area and interaction, which significantly enhance the PTT conversion capacity and O^2^ production, thus improving antibacterial efficacy [24].

Researchers prepared NIR responsive coatings, the most widely used light-responsive type to promote osteogenesis and prevent infection. All these coatings [23, 26, 30, 31, 33–37, 39, 41] (Table 2) have the NIR-responsive ability to produce the PTT effect. For example, Yang et al. reported Au nanorods possess a strong long-wavelength longitudinal plasmon resonance under the range of light from the visible to NIR region. LSPR effects entail Au nanorods have a high light-to-heat conversion ability. Immobilizing Au nanorods as heating source on Ti surface shows a potential application in long-term antibacterial system. Zhang et al. [62] applied curcumin (Cur)/hyaluronic acid (HA)/BMP-2 to the internal TNT doping, which could remove the pathogenic biofilm under NIR irradiation, and Cur reduced the immune response. BMP-2 improves osteogenic differentiation, which accelerated new bone forming. This multifunctional design shows antibacterial and osteogenic functions. Mao et al. [143] reported calcium titanate (CTO) fibrous red phosphorus (RP) on titanium implant surface (Ti‐CTO/RP) and established the P–N heterojunction and internal electric field at the heterointerface to improve the efficiency of charge separation and transfer. NIR excited electron–hole pairs boosts the photocatalytic eradication of MRSA biofilms by ROS. Ti‐CTO/RP can upregulate the expression of bone related genes including lkaline phosphatase (ALP), collagen type I (COL I), osteocalcin (OCN), osteopontin (OPN), osterix (OSX), and Runx-2 to enhance proliferation and differentiation of BMSCs.

**Table 2:** Photoresponsive nanotechnology designs

Response system	Response point	Light	Mechanism	Condition	Preparations process	Results	Bacterial strains
TiO_2_/MoSe_2_/CHI (Chai2021 [21])	TiO_2_	NIR, 808 nm, 0.6 W/cm^2^	PDT, PTT	15 min	1. Preparation of TiO_2_ by MAO 2. Preparation of TiO_2_/MoSe_2_ coating 3. Preparation of TiO_2_/MoSe_2_/CHI	The coating shows antibacterial and osteogenic capability	*S. mutans*
Ti/Phthalocyanines/liposome, nanoemulsion (Faria2014 [22])	Phthalocyanines	Infrared light: 830 nm, 1.5 J, 60 mW Visible light: 633 nm, 3 J, 58 mW	PDT	Infrared light 2 min Visible light 3 min 45 s	1. Liposome preparation 2. Preparation of Oil in Water nanoemulsion for controlled release of chlorine and aluminium phthalocyanine 3. Animal surgery: (a) create defects. (b) Install the implant. (c) Liposome/bone graft/BC/nanoeulsion fill 4. Treatment with visible and infrared light	The use of photosensitivity phthalocyanines activated by LED demonstrated a tendency to stimulate bone formation	–
Bi_2_S_3_@Ag_3_PO_4_/Ti (Hong2019 [24])	Bi_2_S_3_	NIR, 808 nm	PTT, PDT	15 min	1. Preparation of 4-MBA-treated Ti plate by alkali heat 2. Preparation of Bi_2_S_3_/Ti 3. Preparation of Bi_2_S_3_@Ag_3_PO_4_/Ti	PTT and PDT effects break the biofilm	*S. aureus*, *E. coli*
Au/Pt/TiO_2_ (Moon2021 [25])	TNT	Visible light: 470 nm, 660 nm, 5.5 mW/cm^2^		15 min	1. Preparation of TiO_2_ by EA 2. Preparation by Au/Pt-TiO_2_ nanotubes and Pt/Au TiO_2_ nanotubes	Au/Pt can extend the limited UV antibacterial effect and improve the osteogenic perfoormance	*S. aureus*
N-doped TNT (Oh2013 [26])	N-TNT	Visible light: 470 nm, 1000 mW/cm^2^			1. Fabrocation of TNT through EA 2. Fabrocation of N-doped TNT		
PDA-NP-Ti (Ren2020 [28])	PDA	NIR/808 nm, 1 W/cm^2^	PTT	10 min	1. Preparation of PDA-NPs 2. PDA-NPs loaded on Ti	The photothermal PDA-NPs coating shows killing of bacteria and challenging the protective tissue depends on the immersion and acting time	*S. aureus*
RP-IR780-RGDC (Tan2018 [29])	RP, IR780	NIR/808 nm	PTT/PDT	50 °C/10 min	RP film was prepared on Ti surface by CVD PDA modified RP coating RGDG and PDA are loaded on RP membrane by Michael addition reaction	PPT/PDT effects remove the biofilm RGDG improve cell adhesion, proliferation and osteogenic differentiation	*S. aureus*
Ag/CHI@MnO_2_–Ti (Wang2019 [30])	MnO_2_	NIR/808 nm	PTT	20 min	1. MnO_2_-nanosheets were hydrothermally prepared on Ti plates 2. Preparation of CHI/Ag composites with different contents of AgNPs 3. Ag/CHI@MnO_2_–Ti obtaining through electrostatic adsorption	The coating exhibit potential in deep site disinfection of Ti implant through the synergy of pre-releases Ag ions and photothermal effect within a short time	*S. aureus*, *E. coli*
GO-Ag-collagen (Xie2017 [31])	GO, Ag	Visible light/660 nm	PDT	20 min	1. Preparation of GO/AgNPs composites 2. Preparation of GO/AgNPs/collagen hybrid coating on Ti	ROS production and Ag^+^ release shows antibacterial effect	*S. aureus*, *E. coli*
Au-SPR/TiO_2_ (Xu2015 [32])	Au-SPR	Xenon light λ > 420 nm, 50 mWcm^−2^	The hydrophobic and alkyl chains	10 min	1. TNT was prepared through EA AuNPs loaded in TNT ODPA was attached to the tube walls AMP loaded in bottom of TNT	Visible light acts as a touch switch which release drug in TNT to kill bacteria	*E. coli*
β-FeOOH/TiO_2_ coatings (Xue2021 [66])	FeOOH/Fe_2_O_3_	NIR/808 nm, 0.5 Wcm^−2^	PTT	7 min	β-FeOOH/TiO_2_ coatings preparation by micro-arc oxidation	PPT effects remove the biofilm	*S. aureus*
Au nanorods coating (Yang2019 [144])	Au	NIR/808 nm, 0.5 W/cm^2^	PTT	20 min	Preparation of Au nanorods coating on Ti according to electrostatic surface self-assembly technique	The coating shows repeated photothermal antibacterial ability	*E. coli*, *P. aeruginosa*, *S. aureus*, *S. epidermidis*
MoS_2_/PDA-RGD (Yuan2019 [80])	MoS_2_	NIR/808 nm, 0.5 Wcm^−2^	PTT	8 min	MoS_2_ + PDA + RGD coating on Ti	1. Improved the osteogenic ability of BMSCs 2. Effective antibacterial ability under NIR radiation	*S. aureus*, *E. coli*
Ti-M-I-RGD (Yuan2019 [193])	MPDA NPs	NIR/808 nm, 0.5–1.0 Wcm^−2^	PDT/PTT	50 °C/0–10 min	Preparation of MPDA NPs via a one-potsynthesis method Obtaing amino-modified titanium named as Ti–NH_2_ MPDA loaded on Ti–NH_2_ named as Ti-M Covalently fixed RGD on Ti-M named as Ti-M/RGD ICG loaded on Ti-M/RGD by π–π staking reaction named as Ti-M-I-RGD	PTT/PDT effects kill the bacteria	*S. aureus*
FYH/Cur/HAd/BMP-2 NRs (Zhang2021 [62])	NRs	NIR/1060 nm	PTT	45℃/15 min	Preparation of TiO_2_ NRs Preparation of TiO_2_: FYH NRs by load Ho and Yb Preparation of TiO_2_: FYH/Cur/BMP-2 NRs by functionalize TiO_2_: FYH NRs with Cur, HA, BMP-2	Eliminate biofilms on Ti Cur mitigates the immune response. BMP-2 improves osteogenic differentiation, accelerating new bone formation	*S. aureus*
CuS-NP-rGO/TNT coatings (Zhang2021 [176])	CuS, rGO	NIR/808 nm, 2 Wcm^−2^	PTT	10 min	1. TNT preparation by electrochemical anodic oxidation on pure Ti 2. CuS@BSA coatings preparation on TNT by LBL 3. CuS@BSA/rGO-PDA coatings preparation	PPT effects remove the bioflim	*S. aureus*, *E. coli*
CS/Ag/MoS_2_ Ti (Zhu2020 [81])	Ag/MoS_2_	Visible light: 660 nm, 0.898 W/cm^2^	PDT	20 min	1. MoS_2_ were hydrothermally prepared on Ti plates 2. Ag loaded on MoS_2_–Ti 3. CS loaded on Ag/MoS_2_–Ti	1. Ag^+^ reduced the recombination ratio of electron–hole pairs, which enhance the photocatalytic activity of the system 2. CS reduced the cytotoxicity to cells and improve the antibacterial ability	*S. aureus*, *E. coli*

Table 2 is used to show the photoresponsive nanotechnology designs which should be placed in 2.2 photoresponsive strategy part

In addition, the visible light responsive strategy has also been studied in recent years. Faria et al. [22] prepared the liposome complex containing photosensitive phthalocyanines which could stimulate bone formation after activated by light-emitting diode. Besides, Giannelli et al. [23] proved that diode laser (808 nm) can treat peri-implantitis effectively. Oh et al. fabricated nitrogen-doped TNT which showed excellent photocatalytic activity by visible light irradiation [26]. Xie et al. coated photocatalytic AgNPs on rGO nanosheets, coated with proteins to reduce biological toxicity, and irradiated with visible light at 660 nm, showing strong antibacterial properties [31]. Shuang et al. designed Au and Pt NPs coatings on the surface of TiO_2_ nanopillar arrays through SILAR method which improved the photocatalytic performance of TiO_2_ in the visible wavelength region [120].

Photoresponsive agents widely used include noble metal nanostructures (such as silver nanospheres, gold nanorods, etc.) [144], red or black phosphorus [29], carbon-based nanocomposites (such as graphene oxide) [31], sulfide [81], TNT [32], etc. These materials have good photoresponsive effect, but the possibility of burning normal tissues is necessary to be considered in application. The stability of phosphorus needs to be solved, the preparation process of carbon matrix composite coating is complex, the biocompatibility of metal sulfide needs to be paid attention to.

### PH responsive strategy

PH response refers to the application of pH-sensitive materials which can respond the pH changes of the surrounding environment quickly and realize drug on-demand release, which has important application value in biomedical field. PH varies in specific physiological or pathological states. The pH decreases from 7.4 to 5.5 gradually of environment surrounding implant when bacterial infection exists [10]. This change has been applied to design a switch to control the release of antibacterial drugs on different pH-responsive materials. At present, pH-responsive materials include hydrazone bond [42], acetal connector (AL) [41], silk protein [145], etc.

Combining the pH-sensitive molecules with drug-loading nanoparticles or TNT can realize the drugs release in pathological acidic environment, such as triclosan, ibuprofen, vancomycin, antibacterial metal ions, etc. Liu et al. [146] prepared a mixed-shell-polymeric-micelles (MSPM) composed of a hydrophilic PEG shell and a pH-responsive β-amino ester (PAE), loaded with a hydrophobic antibacterial agent triclosan. The MSPM coordination bond is sensitive to low pH environment. Wang et al. [40] combined 1,4-bis (imidazol-1-ylmethyl) benzene (1,4-BIS (Imidazol-1-ylmethyl) benzene, BIX) and metal ions (Zn^2+^ and Ag^+^) to form CPs nanoparticles, which also have pH-responsive coordination bonds. Zhang et al. [36] coated polylysine (PLL) and Cu^2+^ on the surface of TNT loaded with alendronate drugs. Amino modified Cu^2+^ can response pH varying and release drugs. Xiang et al. [39] encapsulated vancomycin (VAN) in TNT, and coated pH sensitive ZnO QD bound with folic acid (FA) on the surface. And drugs can be released at low pH in infectious environment. Dong et al. [41] developed a pH-sensitive AgNPs delivery system by using low pH-sensitive AL and TNTs. Drug release in acidic environments is about 2.5 times higher than in neutral environments. At the same time, the experiment was further discussed. In clinical conditions, the change of pH was not sudden, but gradually occurred with the increase of microbe. This experiment evaluated the release dynamics in the pH 7 range which showed that pH 4–5.5 was more representative of the pH of the infectious environment in clinical conditions. Sang et al. [145] reported gentamicin-silk protein (GS-Silk) coatings which can load drugs on Ti effectively and can achieve intelligent drug release in acidic environment.

Additionally, researchers designed materials sensitive to alkaline environment to prevent sudden release of drugs in acidic environment [37]. Zhou et al. [37] implanted polymer micelles into pH responsive LBL membranes, embedded negatively charged tobramycin (TOB) and positively charged CHI. The micelles loaded with TOB showed rapid release below pH 7.4 and slow release under weakly acidic conditions. It can effectively inhibit initial adhesion and destroy biofilm formation and show a long-term release pattern under acidic conditions to achieve long-term resistance to infection.

In addition to anti-infection studies, some scholars also applied pH-responsive technology to promote bone regeneration around implants. Cheng et al. [42] prepared double-layer nanoparticles which is composed of polylactic acid inner layer and CHI outer layer with pH-sensitive function. Osteoprotegerin (OPG) binds to the CHI skeleton by pH-sensitive hydrazone bond. With the decrease of pH in pathological environment, the cumulative release of OPG increased significantly, which can inhibit the formation of osteoclasts, recruit BMSCs, and promote them differentiate to osteoblasts. At the same time, the inhibitory effect on osteoclasts increased with the decrease of pH (Table 3).

**Table 3:** PH responsive nanotechnology designs

Response system	Stimulus type	Preparation process	Response point	Drug release	Bacterial strains	Antibacterial principle
MSPM-TCS (Liu2016) [146]	Acid Enzyme	MSPM is composed of hydrophilic PEG and β-amino ester PAE	PAE	Low pH sensitivity, targeted to bacterial surfaces Bacteria secrete lipase to degrade PAE and achieve controlled drug release	*S. aureus*	Enhanced physical penetration Targeting in acidic environments Antimicrobial activity
TNT-AL-AgNPs (Dong2017 [41])	Acid	1. Preparation of TNT by anodic oxidation of Ti, COOH-functionalized TNT 2. AL connection of TNT 3. The AgNPs loaded	AL	AL was sensitive to low pH and dissociates at low pH and releases AgNPs inside TNT	*S. aureus* *E. coli*	Antimicrobial activity
TiO_2_-PLL-Cu-NaAL (Zhang2018 [36])	Acid	1. Preparation of TNTs by anodic oxidation of Ti 2. Modified PLL and amino modified Cu^2+^ were loaded on TNT to prepare the coordination system 3. NaAL load of TNT	Coordinate bond	Under low pH or acidic conditions, the system coordination bond is broken, releasing TNT internal drugs	–	–
TNT-CPs (Wang2017 [40])	Acid	1. CPs was prepared by BIX combining with metal ions (Zn^2+^ and Ag+) 2. TNT was prepared by anodic oxidation of Ti 3. Amino functionalized TNT into TNTs 4. IBU, VAN and silver nitrate are loaded into TNTs 5. The CPs TNTs sealing side	Coordinate bond	Under low pH or acidic conditions, the system coordination bond cleavage, CPS destruction, release TNT internal drugs	*S. aureus* *E. coli*	Antimicrobial activity
Ti-PD-(HET/CHT) (Zhou2018 [37])	Alcali	1. Preparation of Tob supported micelles 2. PD coating Ti surface 3. HET/CHT layer by layer assembly to Ti-PD	Micelle	In alkaline environment, the amino Tob is deprotonated, the electrostatic interaction between Tob and heparin is reduced, and the drug is released	*S. aureus* *E. coli*	Antimicrobial activity Inhibition of initial adhesion
TNT-ZnO QD-FA-Van (Xiang2018 [39]	Acid	1. TNT was prepared by anodic oxidation of Ti 2. Modified ZnO QD by FA 3. Van TNT load 4. ZnO QD-FA encapsulated TNT	ZnO	ZnO QD was sensitive to low pH, and the ZnO QD-FA envelope was opened and the drug was released in acidic environment	*S. aureus*	Antimicrobial activity

PH-sensitive materials can achieve high mobility and flexibility, but sudden release of drugs is difficult to achieve long-term effects under the condition of limited drug dosage.

### Enzyme responsive strategy

At different stages of biofilm formation, pathogens secrete different enzymes that can degrade different natural or synthetic polymer molecules. Because of the specific biological binding properties, the incorporation of these polymers into nanocarriers can trigger the drug release reaction at the initial stage of infection and achieve the early control of infection [10]. In addition to anti-infection, some scholars have applied this enzyme response principle to the study of promoting osteogenesis.

At present, bacterial secretase mainly used include hyaluronidase (HAase) [44], Glutamyl endonuclease (V8 enzyme) [46] and chymotrypsin. HAase degrades HA, V8 degrades polyglutamate (PG). Loading these degradable molecules onto TNT nanotubes or nanoparticles is the most widely used design strategy. These effects are applied to design modified implant surfaces with different enzyme-responsive DDS. Including preparation of PG-CHI-Ag-PDOP [46], CHI-Hac-Van [43], Ha-Gen-Chi [44] and Cl13K-MMP9-CP [45] coatings on TNT surface, to achieve accurate specific response ability of antibacterial and osteogenic ability. In addition to coatings, enzyme-responsive nanoparticles are also designed to achieve these functions. Bourgat et al. [47] proposed an enzyme-reacted nano-gel, which combined ciprofloxacin (CIP) with enzyme digestion peptide sequence PLL to form nanoparticles. Under the action of trypsin, the nanoparticles lysed, and CIP released to achieve antibacterial effect (Table 4).

**Table 4:** Enzyme responsive nanotechnology designs

Response system	Acting site	Preparation process	Drug release	Bacterial strains	Result
TNT-DFO-HA-Gen coating (Yu2020) [44]	HAase-HA enzyme	1. Electrochemical treatment of Ti to form TNT 2. Connect HA to Gen 3. Assembly of HA-Gen and Chi 4. DFO is loaded on TNT 5. Ha-gen-chi seal DFO-Ti surface	HA on TNT surface recognizes HAase enzyme secreted by bacterial to achieve specific touch release	*S. aureus* *E. coli*	1. Inhibit bacterial adhesion 2. Antibacterial drug release 3. Promote osseointegration
TNT-Van-Chic-HAc coating (Yuan2018) [43]	HAase-HA enzyme	1. Electrochemical treatment of Ti to form TNT 2. TNT coated Van 3. Dop modified HA-C and Chi-C 4. LBL assembly of HA-C and Chi-C 5. Dop-hac-Chic film is attached to the surface of TNTS-VAN	HA-c on TNT surface recognizes HAase enzyme secreted by bacterial to achieve specific touch release	*S. aureus*	1. Inhibit bacterial adhesion 2. Antibacterial drug release 3. Promote osseointegration
PG-PAH LBL@CHI-Ag coating (Ding2019) [46]	V8 enzyme-PG	1. AgNPs were wrapped in CHI 2. PG and PAH were assembled on CHI-Ag surface by LBL 3. LBL@CHI-Ag was deposited on the surface of Ti matrix modified with PDOP	PG on TNT surface recognizes V8 enzyme secreted by bacterial to achieve specific touch release	*S. aureus*	1. Inhibit bacterial adhesion 2. Antibacterial drug release 3. Promote osteogensis
Ti-GL13K-MMP-9 coating (Fischer2021) [45]	MMP9-CP enzyme-MMP-9	GL13K antimicrobial peptide and MMP9-CP were co-fixed on the surface of Ti	MMP-9 on TNT surface recognizes MMP-CP enzyme secreted by bacterial to achieve specific touch release	*S. gordonii*	1. GL13K antimicrobial peptides antibacterial 2. Promotes bone formation
Ti-CIP-PLL-Alg coating (Bourgat2021) [47]	Trypsin-PLL	1. CIP combine PLL 2. CIP-PLL mixed with Alg to form nanogel 3. Nanoparticles are coated on TI surface	PLL on TNT surface recognizes trypsin enzyme secreted by bacterial to achieve specific touch release	*S. aureus*	Antibacterial drug release

### Electric-responsive

The application of electric-responsive technology on Ti implant surface mainly forces on electrically controlled drug eluting coatings. With the conductive polymers, such as polyaniline, polypyrrole (PPY), TNT eta. can realize drug-controlled release by electrical signals [33–35, 147].

Shi et al. embedded Van in CHI gel. Under the biasing positive voltage to the CHI coated titanium, the CHI gel disintegrates and liberates Van [34]. The deposition time and the applied voltage can influence the amount of drug loading and the rate of drug-elution. TNT has been found to be weak in conductivity, limiting the combination of drug release function and electrical stimulation therapy. In the study of Gulati et al. [35], TiO_2_ formed by anodic oxidation of Ti was converted to titanium nanotubes etaining the tubular structure of TiO_2_ by magnesium heat. Taking Rhodamine B (RhB) as drug model, the drug loading and drug release of Ti NTs showed no significant difference compared with TNT. Bare Ti and nanotube modified Ti implants can be used as electrodes to accept electrical stimulation showing a potential use of enhancing osteoblast and antibacterial functions. Sirivisoot et al. [33] deposited antibiotics and anti-inflammatory drugs dexamethasone (Dex) into PPY (used in corrosion protection, electrochemical biosensors, electrode coatings and bioelectronics as a thin film on conductive materials) on titanium surface, which could release drugs under electric stimulation and potentially lead to alleviate inflammation, promote bone formation, and restrict fibroblasts invasion.

All these experiments confirmed the feasibility of Ti implant surface electrical stimulation responsive drug delivery system. However, no experimental study has been carried out in vivo. The applicational conditions need to be verified. In addition, electric stimulation has good performance on bone tissue healing and regeneration and has been widely used in clinical treatment. However, the limitations like cell damage and poor tissue penetration should be considered in future investigation. Experimental research in vivo based on Ti implant electric-responsive nanotechnology also needs further exploration.

### USW responsive strategy

USW refers to mechanical waves with a wavelength of less than 2 cm, which can penetrate human tissue and focus into a target region. USW could act on the USW-responsive materials as target site and break them, including the polymer micelles, liposomes, hydrophobic air layer etc. Based on the above properties, the ultrasonic-responsive local drug release systems could be built efficiently.

Aw et al. [19] creatively proposed an ultrasonic-triggering drug release system on TNT. TPGS micelles loading indometsin loaded into TNT/titanium implant and placed in phosphate buffered saline solution of pH 7.2 with a sonar probe inserted into the medium, and then USW-mediated drug-micelles release. The USW triggers enhance the penetration of drug complexes into adjacent tissues which are critical for drug delivery to lesion or trauma tissues. Zhou et al. [20] developed a local drug delivery system consisting of superhydrophobic TNT arrays and ultrasonic controlled release triggers. The hydrophilic TNT array is transformed into superhydrophobic array according to treated by 1*H*,1*H*,2*H*,2*H*-perfluorooctyl-triethoxysilane. The surface of the air trapping layer formed in the liquid environment, which showed good isolation effect without additional sealing treatment. Drugs loaded internal can be dissolved by after the trapped air layer was selectively removed by USW (Table 5).

**Table 5:** Electric, USW, RF and USW/NIR stimuli-responsive nanotechnology designs

Stimulus type	Response system	Character	Preparation process	Drug release	Result	Bacterial strains
RF	TNT-AuNPs [60]	1 GHz RF field	1. TNT was prepared by anodic oxidation 2. TNTs loaded AuNPs	AuNPs receive RF stimulation as energytrans inducers	Realize the controlled release system under the RF trigger	–
Electricity	Ti-MWNT-PPy[P/S]-PPy[Dex] [33]	Cyclic voltammetry (CV) 100 mV/s, from − 1 to 1 V for up to 25 cycles	1. Anodized multi-walled carbon nanotubes (MWNTs) on Ti surface 2. Polypyrrole (PPy) is negatively loaded into MWNT 3. Penicillin + streptomycin and DEX were electrodeposited on PPy	PPy[P/S] and PPy[Dex] are electrically stimulated	1. Realize the controlled release system of electrically triggered drugs 2. Anti-bacterial infection 3. Promote bone growth and reduce fibroblast function	–
Electricity	Ti-Chi-Van [34]	Voltages vary between 2–3.5 V	Chi-Van Co-deposited on the surface of titanium plate	Under the stimulation of anodic oxidation, the OH^−^ consumed, pH on titanium surface decreased, and the chitosan gel expanded and decomposed	Anti-bacterial infection	*S. aureus* *E. coli*
Electricity	TNT-Ti [35]	10 V was applied for 1 min (3 cycles, separated by 10 min). 1–15/min	1. TNT was prepared by anodic oxidation 2. Ti was prepared by magnesiothermic reduction 3. Load model drug, RhB	Ti NTs (cathode), bare Ti wire (anode)were connected to power supply	Realize the controlled release system of electrically triggered drugs	–
USW	TNT-TPGS-IND [13]	Requency fixed at 30 kHz; a constant power of 100 W; 1–15 pulses/min; 5 min	1. TNT was prepared by anodic oxidation 2. TPGS polymerized micelles loaded with Ind were loaded into TNTS	USW can destroy TPGS micelles	Controlled release of drugs triggered by USW is realized, and drugs in micelles are not affected by USW, and there is no premature release	–
USW	Liposome-rhBMP-2 nanocomplexes [98]	16 MHz, 5 min	Preparation of rhBMP-2 loaded liposomes	USW triggered rh-BMP-2 release from liposomes	Validated lipid nanoparticles can be USW-triggered delivery of rhBMP-2	–
NIR + USW	Ti–S–TiO_2-x_ [48]	NIR 808 nm, US 1.0 MHz, 15 min	1. TNT was prepared by anodic oxidation of Ti surface 2. Ti–TiO_2_ was doped with S element	–	1. Under near-infrared light and ultrasonic treatment, Ti–S–TiO_2-x_ has effective antibacterial efficiency 2. Stable structure performance to achieve long-term effect	*P. gingiva* Aureus
NIR + USW	PSACT/CNPs-ICG [27]	Diode laser: 810 nm, 250 mW, 31.2 J/cm^2^, 1 min USW: 1 MHz, 100 Hz, 1.56 W/cm^2^, 1 min	1. Preparation of CNPs 2. ICG loaded in CNPs	–	Under the PSACT, the CNPs-ICG nanoparticles shows biofilm removal efficient	*A. actinomycetemcomitans* *P. gingicalis* *P. intermedia*

The current application of USW in the stimuli-responsive nanoengineering on Ti implant mainly lies in the drug delivery system. In addition, USW can also cause the movement of substances in tissues and cells, and the energy generated by USW can also be absorbed by tissues and then converted into heat [148]. These effects can trigger physicochemical changes in tissues [149]. Therefore, there is still a great prospect to study the mechanical and thermal effects induced by USW in the anti-infection and osteogenic ability of implants.

### RF responsive strategy

RF is non-ionizing electromagnetic radiation in the range of 3 kHz to 300 GHz, which can penetrate human tissue deeply. The thermal effect in tissues is permeable uniform and lasts for a long time, which can overcome the problems of excessive heat of other waves concentrated in skin, subcutaneous tissues, and adipose tissues [150]. These advantages make it an ideal choice for non-invasive drug delivery. Bariana et al. [60] prepared micelles (tocopherol PEG succinic acid)-indometacin nanoparticles which loaded in the upper part of TNT. AuNPs placed at the blind end of TNT as a trans-inducer of RF energy to develop a RF-triggered drug release system. After receiving the RF, the active AuNPs can trance drug release rapidly. In addition, RF is widely used in the treatment of tumors [151], nerve injuries [152] and other diseases [153] due to its tissue penetration ability. However, there are few research on the Ti implants, which still has further research and analysis. significances.

### Composite responsive strategy

During the complex and dynamic environment present in pathological tissues, single stimulus-responsive strategy shows limitations. Double or multiple stimuli-responsive nanomaterials can respond to various stimuli in internal or external environment. It can combine the ability of different stimuli-responsive materials to achieve better drug delivery and promote osteogenic function.

#### USW/light

Both USW and light stimulation are highly penetrating and non-invasive to tissues. The researchers coated the titanium substrate surface with materials with both ultrasonic and light response, and received stimulation to produce PTT effect, hole effect, or controlled drug release to achieve therapeutic purposes.

**Fig. 4:**
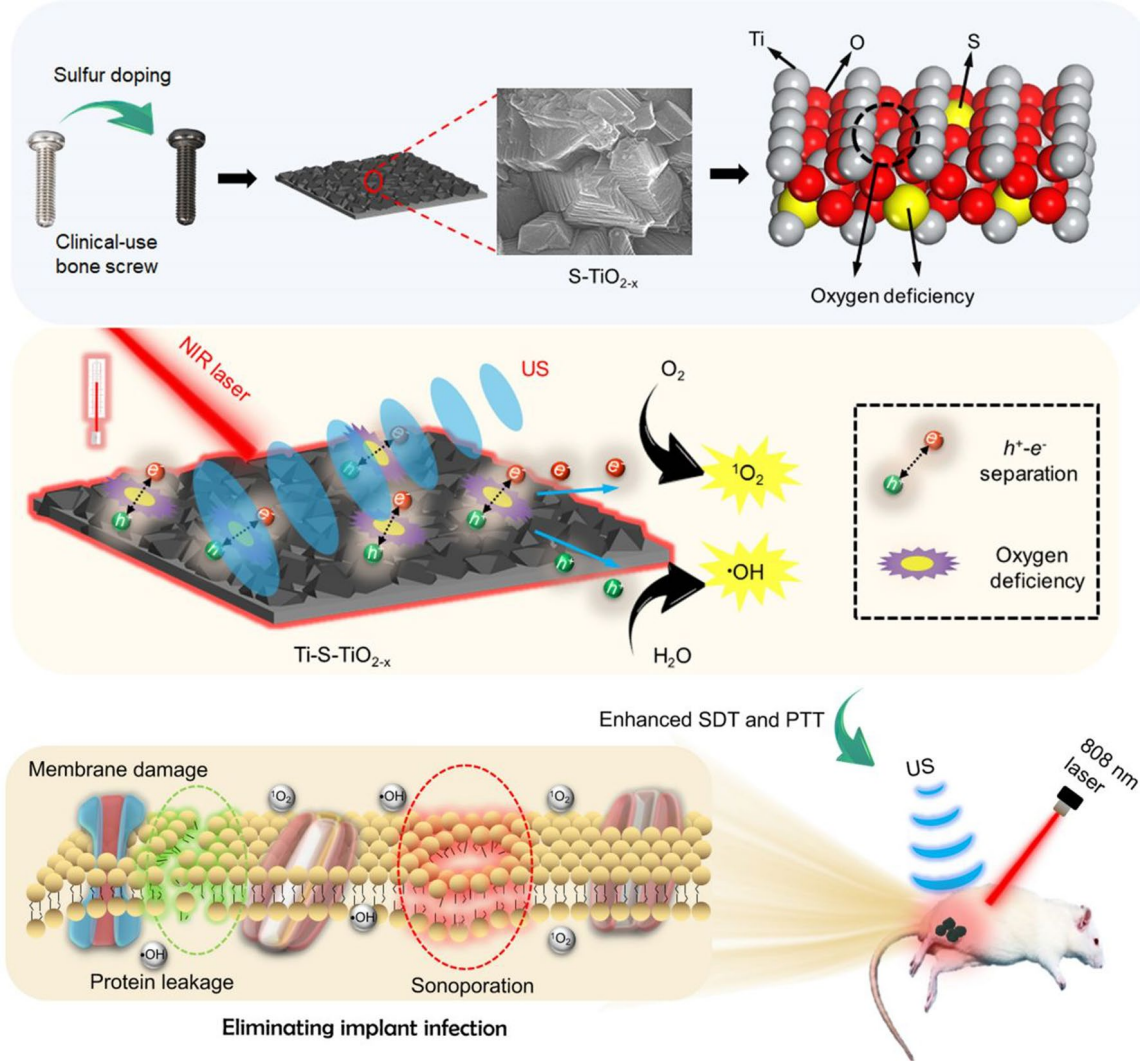
Preparation and morphology of an oxygen-deficient S-doped TiO_2_ layer on the surface of Ti implant (Ti–S–TiO_2-x_) with enhanced sonocatalytic-photothermal properties which exhibits highly effective antibacterial ability and accelerates the osseointegration in vivo. Copyright 2017, ACS Nano

Su et al. [48] prepared a layer of hypoxic S-doped TiO_2-x_ coating (Ti–S–TiO_2-x_) on the surface of titanium implants (Fig. 4). The abilities to response ultrasonic and catalytic PTT properties entail the coating showed high antibacterial ability both in vivo and in vitro under the combined treatment of 808 nm laser and USW. Pourhajibagher et al. [27] targeted sonodynamic antimicrobial chemotherapy (SACT) can bypass the limitations of aPDT and inhibit the characteristics of multimicrobial biofilms. Chitosan nanoparticles (CNPs) indocyanine green (CNPS-ICG) was used as a photoacoustic sensitizer to inhibit the biofilm of pathogens surrounding the surface of titanium implants using antimicrobial photodynamic therapy and SACT, or photoacoustic dynamic antimicrobial chemotherapy (PSACT).

#### pH/enzyme

PH and enzyme-responsive nanosystems can respond directly to the changing physio pathological environment without the need for external stimulation. Therefore, the combination of these two stimulation methods is also widely used in the treatment of tumor and orthopedic diseases.

Liu et al. [146] developed a surface adaptive and pH-responsive nanosystem, which consisting of MSPM, whose coordination bond is sensitive to acidic environment. At physiological pH, the nanoparticles completely penetrate and accumulate in bacterial biofilms due to the stealth properties and negative charge. At low pH near the bacterial cell surface, the nanoparticles become positively charged, thus target themselves with the negatively charged bacterial cell surface, keeping itself in the biofilm and preventing flushing. Once interact with the bacterial cell surface, it is hydrolyzed by bacterial lipase, resulting in the release of the drug.

## Therapeutic applications

**Fig. 5:**
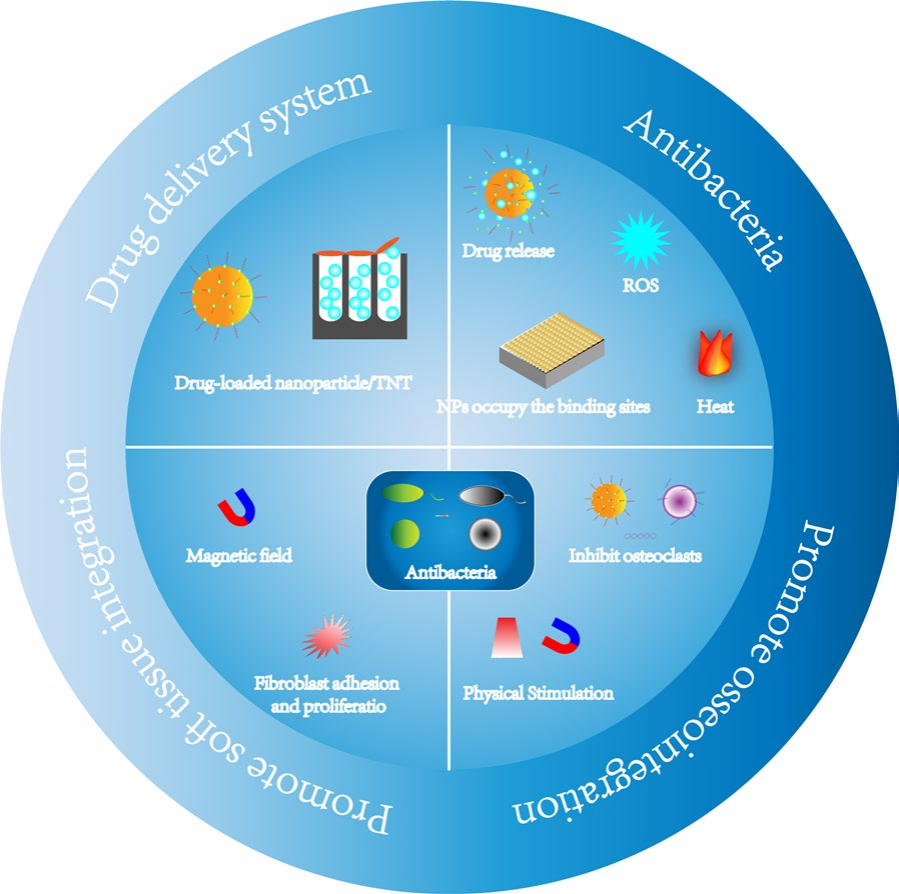
Therapeutic applications of stimuli-response nanosystems

Stimuli-responsive nanotechnologies on Ti implants mainly to obtain better antibacterial and bacteriostasis effects and promote the growth of soft and hard tissues, etc. (Fig. 5). which overcome the limitations and singleness of traditional nanomodifications and can respond to changing physiological or pathological environments flexibly.

### Enhanced antibacterial property

Bacterial infection is the second largest cause of human fatalities which accounts for 17 million patient mortalities every year [154]. Infection is a serious complication of implant-related surgery and is thought to be mainly due to the growth of biofilms on the surface of the implant [155]. Currently, most research has focused on bacterial species which can invade the host and cause various infectious diseases such as *Staphylococcus aureus*, *Helicobacter pylori*, *Pseudomonas aeruginosa*, and *Escherichia coli*. Aseptic technology and systemic antibiotics therapy are mainly traditional methods to prevent infections include but the latter does not often have a very satisfactory outcome [156]. The low effective success rate of systemic antibiotic therapies may be caused by cytotoxicity and side effects to surrounding tissues [157, 158]. Besides, bacterial drug resistance can always be led to by this therapy strategy, but new classes of antibiotic development have been slow [159]. At the same time, with the biofilms gradually formed, antibiotics is often inefficient [160, 161].Therefore, developing efficient antibacterial technology can complete elimination of biofilms without causing bacterial resistance has become important research nowadays.

Stimuli-responsive antibacterial strategy has become a hot spot in the treatment and inhibition of antimicrobial infection in recent years. Stimuli-responsive materials can respond to the changing physical and chemical environment timely and can also be designed for any stage in the formation of biofilms. Stimuli-responsive antibacterial methods are generally classified into two types: (1) preventing bacteria initial adhesion; (2) eliminating the formed biofilms.

The former strategy is mainly used on pH-responsive and enzyme-responsive antifouling surfaces may because pH and enzyme-responsive nanomaterials can respond to the changing physio pathological environment directly and quickly. Molecules like AL, HA, PG, PEG etc. on TNT can occupy sites of implant surface to resist initial bacterial adhesion or kill bacteria by contacting. As far the formed biofilms, it is necessary to eliminate them according to physical and chemical strategies.

The physical strategies contain magnetically mechanical strategy by using magnetic materials pierce the bacteria quickly under a magnetic and high temperature sterilization. All the bacteria cannot resistant the high strength mechanical clear. Cheeseman et al. reported the magneto-responsive gallium based liquid metal (GLM-Fe) nanoparticles formed by Cabrera-Mott oxidation process [162, 163]. In the presence of a rotating magnetic field, the GLM-Fe nanoparticles could be actuated to form different nanoscale-sharp nanoparticles like jagged spheres, nanorods, and nanostars, which average thickness value of edge extending from the sharp GLM-Fe particles is 22.08 ± 17.71 nm [164]. At the same time, under the action of the magnetic field, these magnetic particles rotate at high speed and use their sharp edges to penetrate the biofilm. In addition, heat sterilizations have demonstrated modest antibacterial effects using applications of light pulsing, USW, electric fields, magnetic fields etc. PTT effect and magnetocaloric effect are the most widely used heat production ways. When exposed to light, photon energy interacts with the lattice of PTT sensitive materials. Then the vibration increases, and the temperature increases later causing cell membrane rupture or protein/enzyme denaturation through thermal effects. Based on this effect, the research of PTT antibacterial based on different PTT sensitive materials are also surging. Magnetic nanoparticles around biofilms can generate heat by converting the magnetic loss into heat under the alternating magnetic field [67, 118].

The chemical strategies mainly based on DDS, which can target to the aim tissue or be preloaded on implant. Stimuli-responsive DDS can show flexible controlled release modes, including thermal, magnetic, light, ultrasonic, enzyme, pH, and other types. Including TNT drug delivery system and implant surface coated with drug nanoparticles, and most are local drug DDS. These kinds of DDS deliver drugs at or near the target, with the advantages like improving drug efficiency, reducing the dose required, and reducing toxicity to healthy tissue compared to systematic drug delivery. Drugs like doxycycline, tetracycline, penicillin, cephalexin, gentamicin, chlorhexidine, berberine, daptomycin, levofloxacin, vancomycin, rifampicin, and ions like Ag^+^, Cu^2+^, Zn^2+^, hiding in TNT or nanoparticles can be released and kill bacteria once the infected environment or external stimuli occurred which can avoid a certain extent drug resistance. TNT and nanoparticles are modified with molecules like targeted molecules [51], hydrophilic/hydrophobic molecules [18], biocompatible molecules [149], etc. that respond to different stimuli or improve other properties of nanoparticles according to different materials with drug-carrying properties, such as mesoporous silica [150] and hydrogel [151]. At the same time, complete drug-loaded nanoparticles were prepared by wrapping drugs. These nanoparticles can respond to different stimuli according to the modified components, including light, magnetic, ultrasonic, enzyme, pH, etc. For example, hydrogel-coated gold nanoparticles can respond to light and promote the bone differentiation of human ADSCs [152].

Antibiotics usually inhibit the synthesis of proteins, cell walls and nucleic acids to achieve antimicrobial purpose. Such as doxycycline, tetracycline, gentamicin can inhibit bacterial protein synthesis. Penicillin, cephalexin, and VAN can inhibit cell wall synthesis to achieve antimicrobial effects. Levofloxacin and rifampicine can inhibit bacterial nucleic acid synthesis or function. Chlorhexidine breaks the permeation barrier of cell membranes. In addition to antibiotics, many metal ions also have antibacterial effects on bacteria. Calcium, magnesium and sodium ions can change pH and osmotic pressure to achieve antibacterial effect [75, 165]. Silver, TNT, zinc and copper ions can induce ROS production and damage cell membrane [35, 96, 166]. Additionally, ROS can also be produced by PDT and void effect. At the same time, zinc, zirconium and other metal ions can inhibit the replication of nucleic acid or the interaction between nucleic acid and protein to achieve antibacterial. Gold, on the other hand, can directly destroy the cell wall without producing ROS [167].

Although antibacterial technologies have progressed rapidly, there are still signific issues should be considered of the new antibacterial strategies [168]. The bacterial resistance should be avoided, and biocompatibility and toxicity must be considered carefully [169]. Additionally, targeting capabilities and bacterial responsiveness are promising for the design of antimicrobial materials.

### Improved osseointegration

Scientific research and clinical experience suggest that osseointegration is one of the most important factors to achieve good planting effect [170]. The implant osseointegration could be defined into multiple biological processes including protein adsorption on titanium, inflammatory cell adhesion/inflammatory response, additional relevant cells adhesion, and angiogenesis/osteogenesis [171]. In the processes of osseointegration, the cells around the implantation site secrete various cytokines to promote the recruitment of osteoblasts and induce osteogenic differentiation to achieve bone formation. However, due to lack of good biological activity, the adhesion of osteoblasts on titanium implant materials is poor, and could even result in foreign body reaction, poor local bone quality and slow heal [172]. So, enhancing the bioactivity of implant surface to promote osseointegration is the main strategy to solve these problems. Implant surface topography and composition can affect bioactivity of titanium implant surface. Traditional surface topography improvement methods include sandblasting, acid etching, anodic oxidation and so on [173]. In addition, the performance of extracellular matrix proteins, growth factors and chemokines in improving biocompatibility and promoting bone integration has been confirmed. However, bone progenitor cells and immune cells are in a constantly changing environment. The ability traditional modification to promote bone integration is limited for showing a single function and without ability to respond to changes in different physiological environment. Stimuli-responsive nanotechnology can achieve a good bone-binding effect by improving mechanical properties [174], preventing bacterial infection [175], promoting vascular proliferation, promoting osteogenic behavior of cells and inhibiting the growth of fibroblasts [176] in the changing environments.

According to the response pathways, the surface stimuli-responsive nanotechnologies of Ti implants can be divided into two ways: (1) Stimulation has a benign osteogenic effect to promote osteogenesis in a specific degree by the control of time, strength or other special thresholds [177]. (2) Stimulation triggers drug release or molecular action to indirectly promote osseointegration [178]. Such as anti-inflammatory drugs in responding to stimulation and controlled release of antibacterial properties enhance bone integration.

It has been reported that the moderate external physical stimuli can promote bone integration. Kim et al. [127] reported that moderate intensity SMF can improve osseointegration according to promote proliferation and osteoblastic differentiation of BMSCs, upregulate expression of osteogenic marker genes and increase cell proliferation, ALP activity, calcium release, and mineralized nodule formation. PMF can also accelerate osseointegration by enhance soluble adenylyl cyclase, cyclic adenosine monophosphate (cAMP), protein kinase A, cAMP response element-binding protein signaling and Wingless-related integration site pathway activation [179–181]. According to active the pathways and induce the gene expression, PMF can indirect active of osteoblastic differentiation, proliferation, and activity, antagonize osteoclastic differentiation and activity and enhance osteoblastic differentiation etc. Laser technology has been reported that can enhance osteoblast adhesion and vessel migration towards the implant surface and will not influence implant stability [182, 183]. In electric-responsive technology, conducting polymer can enhance direct electron transfer to promote redox reactions of proteins synthesized by osteoblasts during bone formation [33, 184]. However, most of the studies based on these physical stimuli to promote osseointegration of titanium implants have been conducted in vitro. The clinical outcome still needs more precise design on the types, time, degree of stimuli, and the physical and chemical state of the body.

Research on osteointegration-based stimuli-responsive nanotechnology focuses on local delivery systems. Biological or chemical substances that can promote osteogenesis are encapsulated in nanoparticles or integrated in the implant or scaffolding like TNT, calcium and phosphorus coating, hydroxyapatite, gelatin, CHI, etc. through different methods such as electrospinning, simple coating, physical adsorption, silanization, layer self-assembly technology, etc. are used as carriers. These substances including metal ion, proteins, peptides, growth factors, polysaccharides, and nucleotides. Metal ions like Zn^2+^ and Mg^2+^ can upregulate integrin α1 and integrin β1 gene expression to promote the initial adhesion and spread of rat bone marrow mesenchymal stem cells. Also, they can enhance transcription of Runx2 and expression of ALP and OCN. Upregulation of MagT1 transporter expression in human umbilical vein endothelial cells then stimulates vascular endothelial growth factor and kinase inhibitor transcription through activation of hypoxia-inducible factor-1α, thereby inducing angiogenesis [185–187]. Cu^2+^ can also improve vascularized bone regeneration, bone marrow stromal cell adhesion and proliferation, and subsequent differentiation to osteoblast. The biomolecules like proteins, peptides, growth factors, and nucleotides can involve in biological processes directly. The type I collagen can increase the titanium implant surface bioactivity and accelerate the early osteogenesis [176]. Fibronectin can induce good cellular responses and mediate many cellular processes [188]. Osteogenic growth peptide is a soluble, short and linear growth factor peptide fragment that can directly regulate cell proliferation, osteoblast differentiation and matrix mineralization [189]. Gene fragments like siRNA, miRNA and circRNA have been reported with abilities of improving the surface biocompatibility of titanium implants by promoting the proliferation, differentiation, and adhesion of osteoblasts [190–192]. The stimulation is applied in response to the release of internal biochemicals to achieve good osseointegration.

## Conclusion and prospect

Compared with traditional nanotechnology, stimuli-responsive nanotechnology shows brilliant flexibility, controllability, and specificity, which always combine multiple effects or functions. Both in the application of response types and materials, the research on stimulus response in the field of nanomedicine has made a breakthrough. However, the application of these materials and response types on the surface of orthopedic titanium implants is relatively rare, such as genetic material has received widespread attention which still not be used of the study of titanium implant. In addition, complex stimulus response system is also a research direction should be developed, which has a broad research prospect. Undoubtedly, the goal of various research on surface nanotechnology of titanium implants is to achieve good clinical results, however, most of the studies are in vitro, and more in vivo studies are needed to actively consider clinical outcomes. Currently, most studies concentrate on controlled release drugs by external stimulation, while the stimulating effect of stimulation on soft and hard tissues around Ti remains to be studied like magnetic field, electric field, USW, RF, light and other external stimulation could promote angiogenesis, osteogenesis and osteointegration. These stimulations have made extensive progress in the treatment of tumors and bone defects and have broad application prospects in orthopedic titanium implants. Combining these effects with stimulus-responsive drug delivery system could be a prominent application field in the future.

However, almost none of these smart strategies have reached the commercial market. First, most of the research about stimuli-responsive technologies on titanium implant has been at the in vitro level. Due to the huge differences between in vitro and in vivo experimental studies, and different complex physiological environments between human body and animal models, a lot of in vivo/clinical data is still needed. Secondly, the most appropriate parameters and types of stimuli-responsive materials are still debated. In addition, the nanostructure of the implant surface is very fragile, and the selection of appropriate sterilization, preservation, transportation, and long-term maintenance of material activity are also issues that need to be considered. Lastly, the biggest challenges for the translation market are the clinical trials and their commercial approval by the responsible entities. The commercial translation of implant stimuli-responsive nanotechnologies still has a long way to go.

## Data Availability

Not applicable.

## Abbreviations

Ti: Titanium
ECM: Extracellular matrix
USW: Ultrasound wave
PTT: Photothermal
PDT: Photodynamic
CHI: Chitosan
TNT: TiO_2_ Nanotubes
EA: Anodization
DDS: Drug delivery systems
AuNPs: Au nanoparticles
RF: Radiofrequency
Ind: Indomethacin
ROS: Reactive oxygen species
PEG: Polyethylene glycol
DOP: Dopamine
AgNPs: Ag nanoparticles
ZnO-NPs: Zinc oxide nanoparticles
QD: Quantum dots
CuNPs: Cu nanoparticles
PDA: Polydopamine
RGD: Arginine–glycine–aspartic acid
NIR: Near-infrared light
GO: Graphene oxide
LBL: Layer-by-layer
MAO: Micro-arc oxidation
SILAR: Successive ion layer adsorption and reaction
SMF: Static magnetic fields
PMF: Pulsed magnetic fields
BMSCs: Bone marrow-derived mesenchymal stem cells
ASCs: Adipose-derived mesenchymal stromal cells
ADSCs: Adipose-derived stem cells
MC3T3-E1: Mouse embryo osteoblast precursor cells
SVFs: Stromal vascular fraction cells
MNPS: Magnetic nanoparticles
BMP-2: Bone morphogenetic protein-2
TPGS: d-α-Tocopherol succinate 1000
Cur: Curcumin
HA: Hyaluronic acid
CTO: Calcium titanate
RP: Red phosphorus
ALP: Lkaline phosphatase
COL I: Collagen type I
OCN: Osteocalcin
OPN: Osteopontin
OSX: Osterix
CTO: Calcium titanate
AL: Acetal connector
MSPM: Mixed-shell-polymeric-micelles
PAE: β-Amino ester
BIX: Benzene
PLL: Polylysine
VAN: Vancomycin
FA: Folic acid
GS-Silk: Gentamicin-silk protein
TOB: Tobramycin
OPG: Osteoprotegerin
HAase: Hyaluronidase
PG: Polyglutamate
CIP: Ciprofloxacin
PPY: Polypyrrole
RhB: Rhodamine B
DEX: Dexamethasone
SACT: Sonodynamic antimicrobial chemotherapy
CNPs: Chitosan nanoparticles
PSACT: Photoacoustic dynamic antimicrobial chemotherapy
GLM-Fe: Gallium based liquid metal
cAMP: Cyclic adenosine monophosphate
