# Correction: The current status of stimuli-responsive nanotechnologies on orthopedic titanium implant surfaces

**DOI:** 10.1186/s12951-023-02102-y

**Published:** 2023-10-26

**Authors:** Jingyuan Han, Qianli Ma, Yanxin An, Fan Wu, Yuqing Zhao, Gaoyi Wu, Jing Wang

**Affiliations:** 1 Reconstruction and Regeneration, National Clinical Research Center for Oral Diseases, Shaanxi Engineering Research Center for Dental Materials and Advanced Manufacture, Department of Oral Implants, School of Stomatology, The Fourth Military Medical University, Xi’an, 710032 China; 2 School of Stomatology, Heilongjiang Key Lab of Oral Biomedicine Materials and Clinical Application, Experimental Center for Stomatology Engineering, Jiamusi University, Jiamusi, 154007 China; 3 Department of Biomaterials, Institute of Clinical Dentistry, University of Oslo, 710455 Geitmyrsveien, Oslo Norway; 4 Department of General Surgery, The First Affiliated Hospital of Xi’an Medical University, Xi’an, China

**Correction: Journal of Nanobiotechnology (2023) 21:277** 10.1186/s12951-023-02017-8

Following publication of the author, the affiliation details for the corresponding authors Gaoyi Wu^2^ and Jing Wang^1^ were incorrectly given as:

- ^1^State Key Laboratory of Oral & Maxillofacial Reconstruction and Regeneration, National Clinical Research Center for Oral Diseases, Shaanxi Key Laboratory of Stomatology, Department of Implantology, School of Stomatology, The Fourth Military Medical University, Xi’an 710032, China
- ^2^School of Stomatology, Heilongjiang Key Lab of Oral Biomedicine, Materials and Clinical Application, Experimental Center for Stomatology Engineering, Jiamusi University, Jiamusi 154007, China, but should [1] have been given in this correction.
- ^1^Reconstruction and Regeneration, National Clinical Research Center for Oral Diseases, Shaanxi Engineering Research Center for Dental Materials and Advanced Manufacture, Department of Oral Implants, School of Stomatology, The Fourth Military Medical University, Xi’an, 710032, China
- ^2^School of Stomatology, Heilongjiang Key Lab of Oral Biomedicine Materials and Clinical Application, Experimental Center for Stomatology Engineering, Jiamusi University, Jiamusi, 154007, China

The original article has been revised.
